# Protein *O*-Fucosyltransferases: Biological Functions and Molecular Mechanisms in Mammals

**DOI:** 10.3390/molecules30071470

**Published:** 2025-03-26

**Authors:** Huilin Hao, Benjamin M. Eberand, Mark Larance, Robert S. Haltiwanger

**Affiliations:** 1 Complex Carbohydrate Research Center, University of Georgia, Athens, GA 30605, USA; hl.hao@uga.edu; 2 Charles Perkins Centre, School of Medical Sciences, Faculty of Medicine and Health, The University of Sydney, Sydney, NSW 2006, Australia; bebe0382@uni.sydney.edu.au (B.M.E.); mark.larance@sydney.edu.au (M.L.)

**Keywords:** *O*-fucosylation, POFUT1, POFUT2, POFUT3, POFUT4, FUT10, FUT11, EGF, TSR, EMI, Notch

## Abstract

Domain-specific *O*-fucosylation is an unusual type of glycosylation, where the fucose is directly attached to the serine or threonine residues in specific protein domains via an *O*-linkage. *O*-fucosylated proteins play critical roles in a wide variety of biological events and hold important therapeutic values, with the most studied being the Notch receptors and ADAMTS proteins. *O*-fucose glycans modulate the function of the proteins they modify and are closely associated with various diseases including cancer. In mammals, alongside the well-documented protein *O*-fucosyltransferase (POFUT) 1-mediated *O*-fucosylation of epidermal growth factor-like (EGF) repeats and POFUT2-mediated *O*-fucosylation of thrombospondin type 1 repeats (TSRs), a new type of *O*-fucosylation was recently identified on elastin microfibril interface (EMI) domains, mediated by POFUT3 and POFUT4 (formerly FUT10 and FUT11). In this review, we present an overview of our current knowledge of *O*-fucosylation, integrating the latest findings and with a particular focus on its biological functions and molecular mechanisms.

## 1. Introduction

Fucosylated glycan structures are broadly observed in a wide variety of organisms and play important roles in numerous physiological and pathological processes, including ABO blood group histocompatibility, immune modulation, protein folding, and cellular signal transduction. Two unique features distinguish fucose from other six-carbon sugars: an L-configuration and the absence of a hydroxyl group on the C-6 carbon. Fucose is typically found as terminal or core structures on glycans added by Golgi-localized fucosyltransferases (FUTs), but it can also be directly linked to proteins through an *O*-linkage to the hydroxyl groups of serines or threonines in proteins with specific domains. This domain-specific *O*-fucosylation is mediated by endoplasmic reticulum (ER)-localized protein *O*-fucosyltransferases (POFUTs). A growing body of research has demonstrated the biological importance of *O*-fucosylation. In this review, we will present an overview of our current knowledge of *O*-fucosylation, integrating the latest findings, with a particular focus on its biological functions and molecular mechanisms.

## 2. GDP-Fucose Synthesis and Transport

**Figure 1:**
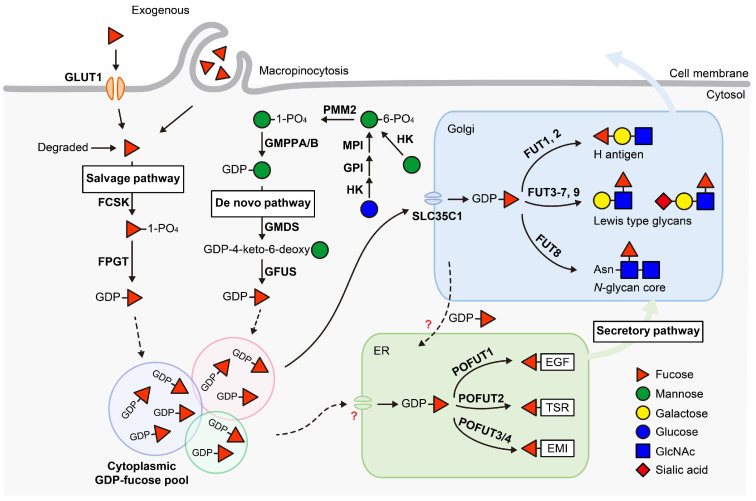
GDP-fucose biosynthesis and transport. GDP-fucose is synthesized in the cytosol via two distinct pathways. The de novo pathway converts mannose or glucose into GDP-fucose, while the salvage pathway utilizes exogenous fucose or fucose salvaged from degraded glycoconjugates. The enzymes responsible for each step are listed and emphasized in bold. GDP-fucose derived from different origins is stored in distinct pools in cytosol and selectively transported into the Golgi by the GDP-fucose transporter *SLC35C1* or into the ER by an as-yet-unknown transporter. The transport of GDP-fucose between the Golgi and ER GDP-fucose pools has been observed, most likely by retrograde transport. Once inside the Golgi or ER, GDP-fucose is transferred to specific substrates by 13 fucosyltransferases (FUTs) in mammals. In the Golgi, nine FUTs (FUT1-9) transfer GDP-fucose to glycan substrates. In the ER, four POFUTs (POFUT1-4) transfer GDP-fucose to proteins containing specific domain structures. These modified proteins are then either secreted or expressed on the cell surface. Dashed arrows indicate trafficking pathways with unknown mechanisms. “?” indicates unknown transporters. HK, hexokinase; GPI, glucose-6-phosphate isomerase; MPI, mannose-6-phosphate isomerase; PMM2, phosphomannomutase 2; GMPPA/B, GDP-mannose pyrophosphorylase A/B; GMDS, GDP-mannose 4,6-dehydratase; GFUS, GDP-L-fucose synthase; GLUT1, glucose transporter type 1; FCSK, L-fucose kinase; FPGT, fucose-1-phosphate guanylytransferase; EGF, epidermal growth factor-like repeat; TSR, thrombospondin type 1 repeat; EMI, elastin microfibril interface domain; FUT, fucosyltransferase; POFUT, protein *O*-fucosyltransferase.

All FUTs utilize guanosine diphosphate (GDP)-fucose as a donor to transfer fucose to acceptor molecules. GDP-fucose is synthesized in the cytosol, then transported into the Golgi or ER, where it is used by Golgi-localized FUTs or ER-localized POFUTs, respectively. In mammals, GDP-fucose is synthesized via two pathways: the de novo pathway and the salvage pathway (Figure 1). The de novo pathway produces GDP-fucose from mannose or glucose, while the salvage pathway generates GDP-fucose from exogenous fucose or fucose salvaged from glycoconjugate degradation.

### 2.1. De Novo Pathway

In the cytosol, glucose or mannose is synthesized into mannose-6-phosphate by a series of enzymes. Mannose-6-phosphate is then converted into mannose-1-phosphate by phosphomannomutase 2 (PMM2) and subsequently synthesized into GDP-mannose by GDP-mannose pyrophosphorylase A/B (GMPPA/B). In the de novo pathway, GDP-mannose that is derived from either mannose or glucose is converted into GDP-fucose. This transformation involves three reactions catalyzed by two enzymes and includes a keto-containing intermediate. GDP-mannose 4,6-dehydratase (GMDS) catalyzes the initial reaction, converting GDP-mannose into GDP-4-keto-6-deoxymannose [1]. This intermediate is epimerized to GDP-4-keto-6-deoxygalactose, then reduced to yield GDP-fucose, with both reactions catalyzed by GDP-L-fucose synthase (GFUS), a dual-functional enzyme acting as both an epimerase and a reductase [2].

### 2.2. Salvage Pathway

In the salvage pathway, the source of fucose is provided by exogenous fucose from the diet or fucose released from the lysosomal degradation of glycoconjugates [3,4]. Free fucose in the cytosol is firstly phosphorylated by L-fucose kinase (FCSK), forming fucose-1-phosphate, and is then converted to GDP-fucose by Fucose-1-phosphate guanylytransferase (FPGT) [5,6]. Exogenous fucose must be transported across cell membranes to present in the cytosol, the mechanism behind this transportation remained largely unknown for decades [7]. Recently, Ng et al., identified GLUT1 (encoded by *SLC2A1*) as a cell membrane fucose transporter using an siRNA screen of ~140 annotated transporter genes in HCT116 cells [8]. They also demonstrated that macropinocytosis plays a role in cellular fucose uptake.

### 2.3. GDP-Fucose Transport and Cellular GDP-Fucose Pool

GDP-fucose synthesized in the cytosol is then transported to the Golgi and ER. The major Golgi GDP-fucose transporter in mammals is *SLC35C1* [9,10]. Lu et al. recently showed that *SLC35C1* knockout in HEK293T cells led to a significant decrease, without complete loss, of O-fucosylation of epidermal growth factor-like (EGF) repeats and thrombospondin type 1 repeats (TSRs) in the ER, implicating crosstalk between the Golgi GDP-fucose pool and the ER GDP-fucose pool [11]. Additionally, *SLC35C1* knockout in mice is perinatal lethal with skeletal defects typical of impaired Notch signaling. *SLC35C2*, a homologue of *SLC35C1*, was predicted to be a GDP-fucose transporter [12]. However, *SLC35C2* knockout in HEK293T cells had no impact on Golgi fucosylation or ER O-fucosylation, and no developmental defects were observed in *SLC35C2* null mice, indicating it is not a GDP-fucose transporter. Although an ER GDP-fucose transporter has been identified in *Drosophila*, its human ortholog, *SLC35B4*, is localized in the Golgi and has been demonstrated as a UDP-xylose/N-acetylglucosamine (GlcNAc) transporter [13]. The mammalian ER GDP-fucose transporter remains to be identified.

The salvage pathway is believed to contribute only a minor portion to the cellular GDP-fucose pool, based on studies in the 1970s that utilized exogenous ^3^H-fucose as a metabolic tracer [14,15]. However, oral fucose supplementation has proven to be an effective therapy for *SLC35C1*-CDG (LAD II) and GFUS-CDG patients with defects in GDP-fucose transportation or de novo synthesis (CDG: congenital disorders of glycosylation) [16]. This suggests that the salvage pathway has the capacity to enhance GDP-fucose production, thus compensating for deficiencies in the transport or de novo synthesis of GDP-fucose. Recent studies revealed that GDP-fucose originating from exogenous sources, salvaged from degradation, or de novo synthesized, is stored in separate and distinct pools rather than a single homogeneous pool [17,18]. By incubating cells with differentially labeled ^13^C-monosaccharides and measuring their contributions to glycans using gas chromatography-mass spectrometry (GC-MS), Sosicka et al. demonstrated that cells identify and selectively use different GDP-fucose pools depending on their heritage [17]. These pools communicate with each other to tightly maintain the total GDP-fucose pool at a constant level. Feedback inhibition of GMDS likely plays a role in this regulatory mechanism, as GDP-fucose is a potent competitive inhibitor for GMDS, allowing efficient fine-tuning of individual pool sizes [19,20,21].

## 3. Fucosyltransferases (FUTs)

**Figure 2:**
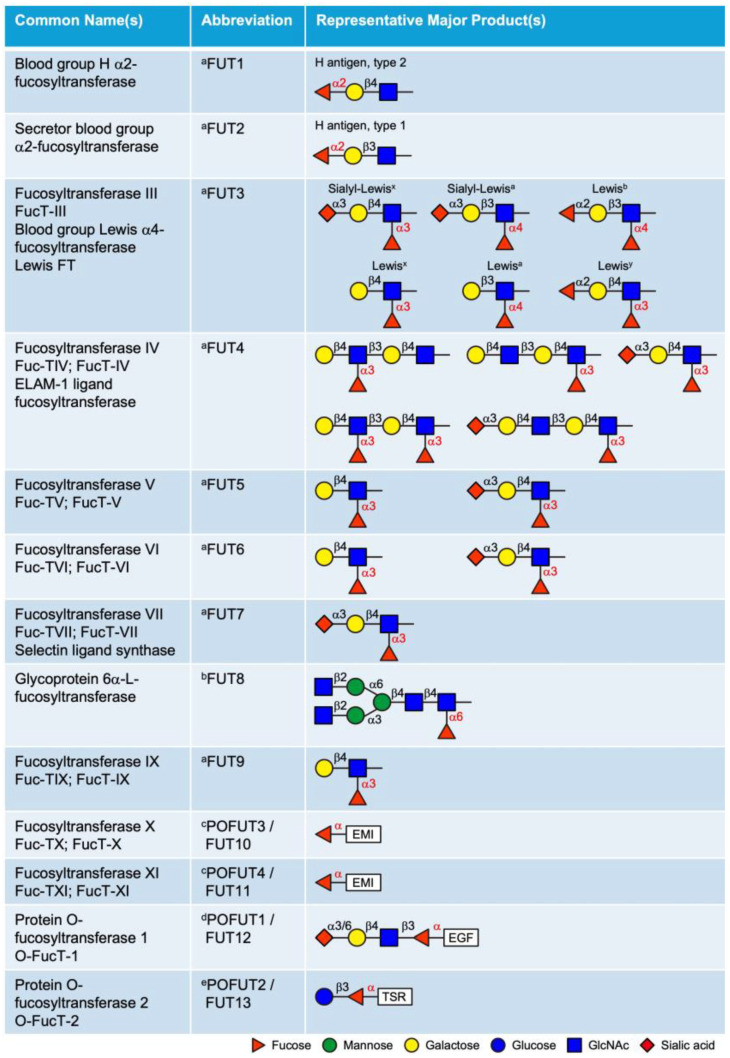
List of 13 fucosyltransferases (FUTs) in mammals and their representative products. The linkage of the fucose added by each enzyme is labeled in red. ^a^ These enzymes add fucose to oligosaccharide chains on glycolipids, *N*-glycans, mucin *O*-glycans or glycoRNAs. ^b^ This enzyme adds fucose specifically to the innermost GlcNAc of the chitobiose unit in *N*-glycans. ^c^ This modification is observed only in EMI domains; see Figure 3C. ^d^ This modification is observed only in EGF repeats with the consensus sequence C^2^XXXX[S/T]C^3^; see Figure 3A. ^e^ This modification is observed only in TSRs with the consensus sequence C^1−2^XX[S/T]C^2−3^; see Figure 3B. EGF, epidermal growth factor-like repeat; TSR, thrombospondin type 1 repeat; EMI, elastin microfibril interface domain. Updated from Schneider et al., 2017 [24].

FUTs catalyze the transfer of a fucose residue from the donor substrate GDP-fucose to acceptor molecules, including oligosaccharides, glycoproteins, glycolipids, and glycoRNAs [22,23,24,25]. To date, 13 FUTs have been identified in mammals based on conserved sequences that are involved in GDP-fucose binding (Figure 2). These FUTs are classified into α2-, α3/4-, α6-FUTs, and POFUTs, based on their distinct fucose linkages. While POFUTs are ER-localized proteins, all other FUTs are type II transmembrane proteins localized in the Golgi. Sequence alignment of the 13 human FUTs identified three distinct groups with high sequence homology: FUT1 and FUT2, FUT3–7 and FUT9 (with particularly high homology among FUT3, FUT5, and FUT6), and FUT10 and FUT11. In contrast, FUT8, POFUT1, and POFUT2 show limited homology with other FUTs (Supplemental Table S1).

### 3.1. Golgi-Localized FUTs

The Golgi-localized FUTs include FUT1–9, all of which transfer fucose to glycan structures as terminal or core modifications. FUT1, FUT2, and Sec1 (non-functional protein encoded by a pseudogene) are α2-FUTs that belong to the CAZy GT11 family [26,27]. FUT1 and FUT2 are responsible for synthesizing the H antigens (O blood), which serve as fucosylated precursors for the A and B oligosaccharide structures in the ABH blood group system [28,29]. They exhibit distinct tissue expression patterns and acceptor specificities. FUT1 product (H antigen) is primarily expressed on erythrocyte membranes and vascular endothelium, with a preference for type 2 structures, whereas FUT2 (Se) is predominantly expressed in epithelial cells and exocrine secretions and is more active on type 1 structures. FUT3–7 and FUT9 are α3/4-FUTs that belong to the CAZy GT10 family, all of which have α3 activity, except FUT3 and FUT5, which also possess α4 activity [30,31,32,33,34,35]. These FUTs are involved in the synthesis of the complex series of Lewis antigen epitopes (i.e., Lewis^x^, Lewis^y^, Lewis^a^, Lewis^b^, Sialyl-Lewis^x^, Sialyl-Lewis^a^) with distinct acceptor specificities. The biological roles of the ABH and Lewis antigens have been previously reviewed in detail [36]. FUT8 is the only α6-FUT identified in mammals, belonging to the CAZy GT23 family [37,38]. FUT8 catalyzes *N*-glycan core fucosylation by transferring a fucose moiety to the innermost GlcNAc residue of the chitobiose unit. Core fucosylation plays critical roles in the regulation of antibody-dependent cellular cytotoxicity (ADCC) and serves as a biomarker for various types of cancer [39,40].

Additionally, based on sequence homology to known α3/4-FUTs, FUT10 and FUT11 were postulated to be α3-FUTs. However, as will be detailed below, substantial experimental evidence confirms that these enzymes function as POFUTs rather than α3-FUTs.

### 3.2. ER-Localized POFUTs

Unlike the Golgi-localized FUTs that transfer fucose to glycan structures, POFUTs add fucose directly to the hydroxyls of serines or threonines of proteins through an *O*-linkage. In mammals, four POFUTs have been identified: POFUT1 (CAZy GT65 family), POFUT2 (CAZy GT68 family), POFUT3 (formerly FUT10, CAZy GT10 family), and POFUT4 (formerly FUT11, CAZy GT10 family) [41,42,43,44,45]. POFUT1 contains a C-terminal KDEL-like sequence that retains it in the ER. While POFUT2 does not contain the ER retention sequence, it is proposed to be retained in the ER by association with other proteins that contain retention sequences. Similarly, POFUT3 and POFUT4 do not contain a KDEL-type ER retention sequence and likely bind to other ER proteins for retention in the ER. All POFUTs mediate domain-specific *O*-fucosylation and are highly specific for their respective substrates [45,46]. Unlike the Golgi-localized FUTs, all POFUTs require a folded domain structure for modification. The binding interface between each POFUT and its respective substrate displays a ‘hand-in-glove’ complementarity, with the substrate domain structure fitting into the enzyme’s groove, precisely positioning the hydroxyl group of serine or threonine for fucose transfer. This will be discussed in detail in Section 5.

#### 3.2.1. POFUT1 (FUT12)

**Figure 3:**
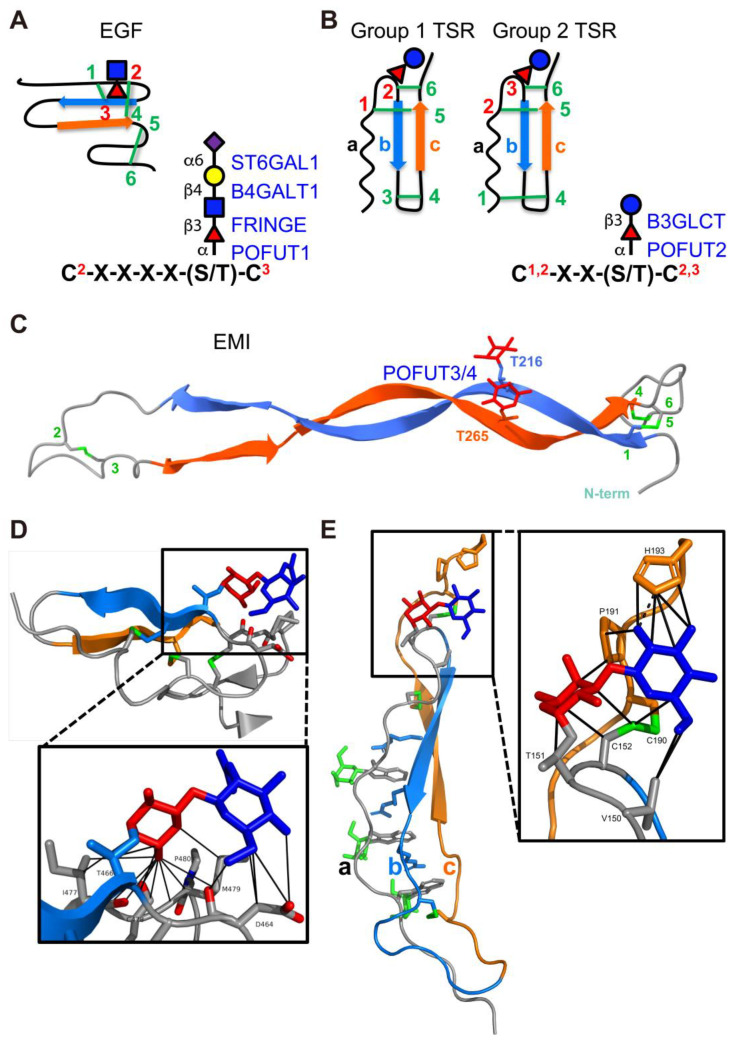
EGF repeats, TSRs, and EMI domains are modified by *O*-fucose glycans. (**A**) (left) Disulfide bonding pattern of an EGF repeat. Cysteines are numbered, disulfide bonds are in green, β-strands are indicated as blue and orange arrows. *O*-fucose site is marked with fucose (red triangle), GlcNAc (blue square), Galactose (yellow circle), and Neu5Ac (purple diamond). (right) Consensus sequence for POFUT1 modification. C^2^ and C^3^ are the second and third conserved cysteine in the EGF repeat. X indicates any amino acid. Enzymes responsible for modification are in blue with linkages indicated. (**B**) (left) Two distinct disulfide bonding patterns for TSRs. Cysteines are numbered, disulfide bonds are in green, β-strands are indicated as blue and orange arrows. *O*-fucose site is marked with fucose (red triangle) and glucose (blue circle). (right) Consensus sequence for POFUT2 modification. The C’s can be C^1^ and C^2^ or C^2^ and C^3^, depending on whether the TSR is Group 1 or Group 2. Enzymes responsible for modification are in blue with linkages indicated. (**C**) AlphaFold2 predicted structure of the EMI domain from MMRN1 showing the disulfide bonding pattern. Cysteines are numbered, disulfide bonds are in green, β-strands are indicated as blue and orange arrows. The two *O*-fucose sites are indicated and marked with fucose residues in red. POFUT3 and POFUT4 are responsible for modification and indicated in blue. (**D**) Structure of NOTCH1 EGF12 modified with a GlcNAcβ1-3Fucose disaccharide (from PDB ID 4D0E). β-strands colored as in (**A**). Fucose in red, GlcNAc in blue, disulfide bonds in green, oxygen atoms highlighted in red. Box shows zoomed in region highlighting interactions of the disaccharide with underlying amino acids identified by MolProbity [79] (van der Waals, solid lines). Structures rendered in PyMOL (version 2.2.2). (**E**) Structure of Properdin TSR2 modified with Glucoseβ1-3Fucose disaccharide (from PDB ID 6RUS). The three strands (a, b, and c) of the TSRs are colored as in (**B**). Fucose in red, glucose in blue, mannose in green, disulfide bonds in green. Box shows zoomed in region highlighting interactions of the disaccharide with underlying amino acids identified by MolProbity [79] (H-bonds, dashed lines; van der Waals, solid lines). Structures rendered in PyMOL (version 3.0.4). Panels A, B and D are modified with permission from Ref. [44].

The unique fucose–protein linkage was initially identified in the urinary-type plasminogen activator (urokinase) using mass spectrometry [47,48]. This *O*-fucose monosaccharide was located on the single epidermal growth factor-like (EGF) domain of urokinase within a sequence C^2^XXGG[S/T]C^3^ (where C^2^ and C^3^ are the second and third cysteine in the EGF, S/T is the modified site). EGF domains are small protein domains with ~40 amino acids, featuring six highly conserved cysteines that form three disulfide bonds (Figure 3A). Soon after, more proteins with *O*-fucose monosaccharide or elongated tetrasaccharide were identified, all containing one or more EGF repeats, including t-PA [49], coagulation factor VII [50], factor XII [51], and factor IX [52]. These discoveries sparked an intensive investigation to identify the enzyme responsible for this modification. The Spellman group developed an enzymatic assay to test *O*-fucosyltransferase activity [53]. Interestingly, using extracts from Chinese hamster ovary (CHO) cells as the enzyme source, they observed activity when employing the complete EGF domain from human factor VII (bacterially expressed, unmodified) as the acceptor substrate, but not with synthetic peptides containing the consensus sequence. This indicates that the enzyme requires a properly folded EGF structure for its activity, which sets it apart from other glycan-modifying FUTs. Using this complete EGF domain from human factor VII (bacterially expressed) as bait, the enzyme (O-FucT-1) was successfully purified from CHO cells by the same group [54]. The sequence of O-FucT-1 was used in a screening of a human cDNA library and led to the identification of the human *POFUT1* gene [55]. Homologues in mice (*Pofut1*), *Drosophila* (*Ofut1*), and *C. elegans* (*pofut1*) were also identified. The *POFUT1* gene is highly conserved in mammals and shows nearly ubiquitous expression in all tissues examined, indicating the potential important biological roles of *O*-fucosylation.

Our knowledge on POFUT1-mediated EGF *O*-fucosylation has largely expanded as more protein targets have been uncovered and increased research efforts on exploring the biological functions. A refined consensus sequence C^2^XXXX[S/T]C^3^ was proposed to include a broader set of *O*-fucose sites [56,57,58]. Database searches with this consensus sequence in the context of an EGF repeat identified more than 100 putative protein targets for POFUT1 (splice variants are not included) (Table 1) [24,59]. However, the *O*-fucosylation status of many of these proteins is yet to be confirmed.

**Figure 4:**
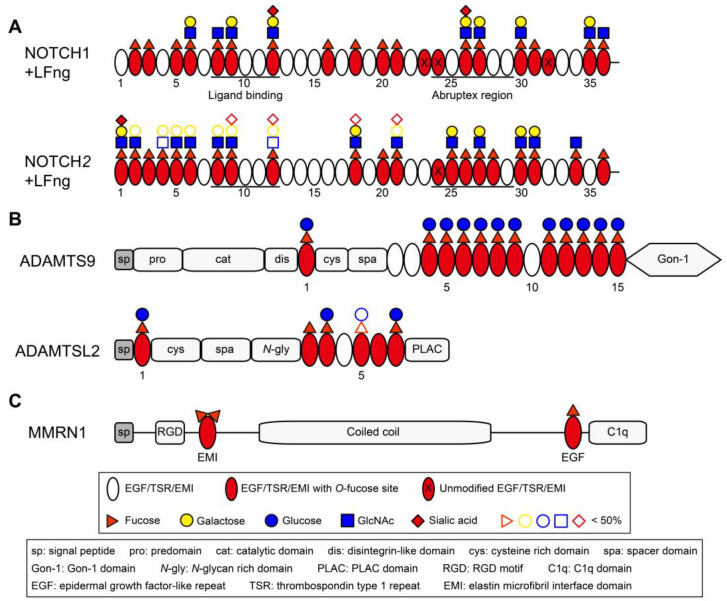
Domain maps of representative EGF/TSR/EMI-containing proteins showing distribution of *O*-fucosylation. (**A**) Domain maps of mouse NOTCH1 EGF1–36 and NOTCH2 EGF1–36. The majority of EGF repeats are modified with *O*-fucose glycans with more than half elongated by Lfng (modified from Ref. [66] with permission). (**B**) Domain maps of human ADAMTS9 and mouse ADAMTSL2. The majority of TSRs in ADAMTS9 and ADAMTSL2 are modified with Glucoseβ1-3Fucose disaccharide by POFUT2 and B3GLCT (modified from Refs. [59,230] with permission) [213]. (**C**) Domain map of human MMRN1 showing the EMI domain at protein N-terminal and can be modified with two fucose residues.

**Figure 5:**
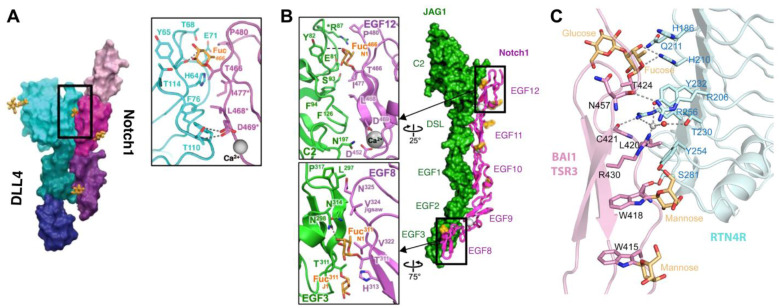
*O*-fucose glycans form direct contacts with binding partners. (**A**) Co-crystal structure of NOTCH1 EGF11–13 (magenta/purple) and DLL4 N-EGF1 (blue/green) (modified with permission from Ref. [72]). Zoomed panel shows direct interaction between fucose residue on NOTCH1 EGF12 with residues in DLL4. (**B**) Co-crystal structure of NOTCH1 EGF8–12 (magenta) and JAG1 N-EGF3 (green) (modified with permission from Ref. [73]). Zoomed panels show direct interactions between fucose residues on EGF8 and EGF12 with residues in JAG1. (**C**) Co-crystal structure of RTN4R (cyan) and BAI1 TSR3 (pink) (modified with permission from Ref. [235]). *O*-fucose forms direct interactions with residues in RTN4R.

Heterozygous mutations of *POFUT1* in human causes a rare skin condition known as Dowling–Degos Disease 2 (DDD2), characterized by skin pigmentation abnormalities [60]. *Pofut1* knockout in mice is embryonic lethal with multiple severe developmental defects, including angiogenesis, hematopoiesis, neurogenesis and somitogenesis. These phenotypes are similar to those observed in *Notch1* knockout mice embryos, indicating Notch as the primary biological target of POFUT1 in embryogenesis [61,62]. The Notch family proteins possess more predicted sites for POFUT1 modification than any other proteins and are the most extensively studied POFUT1 targets (Table 1) [63]. Notch is a key regulator in a variety of developmental processes [64]. *O*-fucosylation of Notch is essential for its proper functioning in processes like the Notch-dependent regulation of lymphopoiesis and myelopoiesis [65]. The extracellular domain of Notch is heavily decorated with *O*-fucose glycans (Figure 4A) [66,67]. A substantial number of *O*-fucose glycans are elongated to GlcNAcβ1-3Fucose disaccharide by the Fringe family of β3-*N*-acetylglucosaminyltransferases, and can be further elongated to a tetrasaccharide, Siaα2-6Galβ1-4GlcNAcβ1-3Fuc [56,67,68,69,70,71]. Interestingly, *O*-fucose glycans on distinct EGF repeats play varying roles in the regulation of Notch activity. *O*-fucose residues on EGF8 and EGF12 of NOTCH1 directly interact with ligands, Delta-like 1 and Jagged1, and their binding is further enhanced by Fringe modifications (Figure 5) [67,72,73,74]. However, Fringe modifications on EGF6 and EGF36 inhibit NOTCH1 activation by Jagged1 [67]. Through the regulation of POFUT1, Fringe, and several other protein *O*-glycosyltransferases associated with Notch, cells can dynamically fine-tune Notch activity to accommodate environmental changes. More details on the effects of *O*-fucose and other *O*-glycans on Notch function can be found in these recent reviews: [65,75,76,77,78].

**Table 1:** List of putative human protein substrates of POFUT1.

UniProt ID	Gene Name	Protein Name	Consensus/Total	Subcellular Location	Known Human Pathology (If Any)
H0YMF1	*ACAN*	Aggrecan core protein	1/2	ECM	-
Q14246	*ADGRE1*	Adhesion G protein-coupled receptor E1	4/6	CM	-
Q9UHX3	*ADGRE2*	Adhesion G protein-coupled receptor E2	2/5	CM	Vibratory urticaria (VBU) [80]
Q9BY15	*ADGRE3*	Adhesion G protein-coupled receptor E3	1/2	ECM, CM	-
Q86SQ3	*ADGRE4P*	Putative adhesion G protein-coupled receptor E4P	1/2	ECM, CM	-
P48960	*ADGRE5*	Adhesion G protein-coupled receptor E5	2/5	ECM, CM	-
**O00468**	***AGRN***	**Agrin**	**2/4** [81]	ECM	Myasthenic syndrome, congenital, 8 (CMS8) [82,83,84]
Q6UW56	*ATRAID*	All-trans retinoic acid-induced differentiation factor	1/1	CM, N	-
Q96GW7	*BCAN*	Brevican core protein	1/1	ECM, CM	-
Q9NPY3	*CD93*	Complement component C1q receptor	1/5	CM	-
Q9NYQ6	*CELSR1*	Cadherin EGF LAG seven-pass G-type receptor 1	2/8	CM	Neural tube defects (NTD) [85]; Lymphatic malformation 9 (LMPHM9) [86,87]
Q9HCU4	*CELSR2*	Cadherin EGF LAG seven-pass G-type receptor 2	2/7	CM	-
Q9NYQ7	*CELSR3*	Cadherin EGF LAG seven-pass G-type receptor 3	2/8	CM	-
P0CG37	*CFC1*	Cryptic protein	1/1	ECM, CM	Heterotaxy, visceral, 2, autosomal (HTX2) [88,89]
P0CG36	*CFC1B*	Cryptic family protein 1B	1/1	ECM	-
Q8WYK1	*CNTNAP5*	Contactin-associated protein-like 5	1/2	CM	-
P82279	*CRB1*	Protein crumbs homolog 1	8/19	ECM, CM	Retinitis pigmentosa 12 (RP12) [90,91]; Leber congenital amaurosis 8 (LCA8) [92]; Pigmented paravenous chorioretinal atrophy (PPCRA) [93]
Q5IJ48	*CRB2*	Protein crumbs homolog 2	9/15	ECM, CM, CYT	Focal segmental glomerulosclerosis 9 (FSGS9) [94]; Retinitis pigmentosa (RP) [95]; Ventriculomegaly with cystic kidney disease (VMCKD) [96]
**P13385**	***CRIPTO***	**Protein Cripto**	**1/1** [97,98]	ECM, CM	-
P51864	*CRIPTO3*	Putative protein CRIPTO3	1/1	CM	-
O60494	*CUBN*	Cubilin	4/7	CM	Imerslund-Grasbeck syndrome 1 (IGS1) [99,100,101]; Proteinuria, chronic benign (PROCHOB) [102]
**P80370**	***DLK1***	**Protein delta homolog 1**	**3/6** [103]	CM, CYT	-
Q6UY11	*DLK2*	Protein delta homolog 2	1/6	CM	-
**O00548**	***DLL1***	**Delta-like protein 1**	**4/8** [56,58]	CM	Neurodevelopmental disorder with non-specific brain abnormalities and with or without seizures (NEDBAS) [104]
**Q9NYJ7**	***DLL3***	**Delta-like protein 3**	**2/6** [105]	CM	Spondylocostal dysostosis 1, autosomal recessive (SCDO1) [106]
Q9NR61	*DLL4*	Delta-like protein 4	5/8	CM	Adams-Oliver syndrome 6 (AOS6) [107]
Q8NFT8	*DNER*	Delta and Notch-like epidermal growth factor-related receptor	6/10	CM	-
**O43854**	***EDIL3***	**EGF-like repeat and discoidin I-like domain-containing protein 3**	**1/3** [108]	ECM	-
O95967	*EFEMP2*	EGF-containing fibulin-like extracellular matrix protein 2	1/6	ECM	Cutis laxa, autosomal recessive, 1B (ARCL1B) [109]
P01133	*EGF*	Pro-epidermal growth factor	1/9	CM	Hypomagnesemia 4 (HOMG4) [110]
Q9UHF1	*EGFL7*	Epidermal growth factor-like protein 7	1/2	ECM	-
Q63HQ2	*EGFLAM*	Pikachurin	2/3	ECM	-
Q5T1H1	*EYS*	Protein eyes shut homolog	11/27	ECM, CYT	Retinitis pigmentosa 25 (RP25) [111,112]
**P08709**	***F7***	**Coagulation factor VII**	**1/2** [50]	ECM	Factor VII deficiency (FA7D) [113,114,115]
**P00740**	***F9***	**Coagulation factor IX**	**1/2** [52,116]	ECM	Hemophilia B (HEMB) [117,118]; Thrombophilia, X-linked, due to factor IX defect (THPH8) [119]; Warfarin sensitivity, X-linked (WARFS) [120]
**P00748**	***F12***	**Coagulation factor XII**	**1/2** [51]	ECM	Factor XII deficiency (FA12D) [121,122]; Angioedema, hereditary, 3 (HAE3) [123,124]
Q14517	*FAT1*	Protocadherin Fat 1	2/5	CM, N	-
Q9NYQ8	*FAT2*	Protocadherin Fat 2	1/2	CM	Spinocerebellar ataxia 45 (SCA45) [125]
Q8TDW7	*FAT3*	Protocadherin Fat 3	3/4	CM	-
Q6V0I7	*FAT4*	Protocadherin Fat 4	5/6	CM	Van Maldergem syndrome 2 (VMLDS2) [126]; Hennekam lymphangiectasia-lymphedema syndrome 2 (HKLLS2) [127]
P23142	*FBLN1*	Fibulin-1	3/9	ECM	Complex type of synpolydactyly [128]; associated with human breast cancer [129]
P98095	*FBLN2*	Fibulin-2	2/10	ECM	-
Q9UBX5	*FBLN5*	Fibulin-5	1/6	ECM	Charcot-Marie-Tooth disease, demyelinating, 1H (CMT1H) [130]; Cutis laxa, autosomal dominant, 2 (ADCL2) [131]; Cutis laxa, autosomal recessive, 1A (ARCL1A) [132]; Macular degeneration, age-related, 3 (ARMD3) [133]
Q53RD9	*FBLN7*	Fibulin-7	2/3	ECM	-
**P35555**	***FBN1***	**Fibrillin-1**	**1/47** [134]	ECM	Marfan syndrome (MFS) [135,136]; Ectopia lentis 1, isolated, autosomal dominant (ECTOL1) [137]; Weill–Marchesani syndrome 2 (WMS2) [138]; Overlap connective tissue disease (OCTD) [139]; Stiff skin syndrome (SSKS) [140]; Geleophysic dysplasia 2 (GPHYSD2) [141]; Acromicric dysplasia (ACMICD) [141]; Marfanoid-progeroid-lipodystrophy syndrome (MFLS) [142]
P35556	*FBN2*	Fibrillin-2	2/47	ECM	Contractural arachnodactyly, congenital (CCA) [143]; Macular degeneration, early-onset (EOMD) [144]
Q75N90	*FBN3*	Fibrillin-3	1/44	ECM	-
Q14520	*HABP2*	Hyaluronan-binding protein 2	1/3	ECM	Thyroid cancer, non-medullary, 5 (NMTC5) [145,146]
Q04756	*HGFAC*	Hepatocyte growth factor activator	2/2	ECM	-
P98160	*HSPG2*	Basement membrane-specific heparan sulfate proteoglycan core protein	3/4	ECM	Schwartz-Jampel syndrome (SJS1) [147]; Dyssegmental dysplasia Silverman-Handmaker type (DDSH) [148]
**P78504**	***JAG1***	**Jagged-1**	**11/16** [56,149]	CM	Alagille syndrome 1 (ALGS1) [150]; Tetralogy of Fallot (TOF) [151]; Deafness, congenital heart defects, and posterior embryotoxon (DCHE) [152]; Charcot-Marie-Tooth disease, axonal, 2HH (CMT2HH) [153]
Q9Y219	*JAG2*	Jagged-2	9/16	CM	Muscular dystrophy, limb-girdle, autosomal recessive 27 (LGMDR27) [154]
Q07954	*LRP1*	Prolow-density lipoprotein receptor-related protein 1	5/22	CM, CYT	Keratosis pilaris atrophicans (KPA) [155]; Developmental dysplasia of the hip 3 (DDH3) [156]
Q9NZR2	*LRP1B*	Low-density lipoprotein receptor-related protein 1B	4/22	CM	-
Q14767	*LTBP2*	Latent-transforming growth factor beta-binding protein 2	1/20	ECM	Glaucoma 3, primary congenital, D (GLC3D) [157]; Microspherophakia and/or megalocornea, with ectopia lentis and with or without secondary glaucoma (MSPKA) [158]; Weill–Marchesani syndrome 3 (WMS3) [159]
Q5VYJ5	*MALRD1*	MAM and LDL-receptor class A domain-containing protein 1	1/1	CYT	-
O75095	*MEGF6*	Multiple epidermal growth factor-like domains protein 6	5/27	ECM	-
Q7Z7M0	*MEGF8*	Multiple epidermal growth factor-like domains protein 8	2/5	CM	Carpenter syndrome 2 (CRPT2) [160]
Q96KG7	*MEGF10*	Multiple epidermal growth factor-like domains protein 10	5/15	CM	Congenital myopathy 10A, severe variant (CMYP10A) [161]; Congenital myopathy 10B, mild variant (CMYP10B) [162]
A6BM72	*MEGF11*	Multiple epidermal growth factor-like domains protein 11	8/14	CM	-
**Q13201**	***MMRN1***	**Multimerin-1** [45,163]	**1/1**	ECM	Factor V Quebec [164]
Q9UK23	*NAGPA*	N-acetylglucosamine-1-phosphodiester alpha-N-acetylglucosaminidase	1/2	GA	Stuttering [165]
A0A087WY62	*NBPF26*	NBPF member 26	5/6	CYT	-
O14594	*NCAN*	Neurocan core protein	2/2	ECM	-
Q92832	*NELL1*	Protein kinase C-binding protein NELL1	1/5	ECM, CYT	-
Q14112	*NID2*	Nidogen-2	1/5	ECM	-
**P46531**	***NOTCH1***	**Neurogenic locus notch homolog protein 1**	**21/36** [67,70]	CM	Aortic valve disease 1 (AOVD1) [166]; Adams-Oliver syndrome 5 (AOS5) [167]
**Q04721**	***NOTCH2***	**Neurogenic locus notch homolog protein 2**	**20/36** [66,168]	CM	Alagille syndrome 2 (ALGS2) [169]; Hajdu-Cheney syndrome (HJCYS) [170]
Q7Z3S9	*NOTCH2NLA*	Notch homolog 2 N-terminal-like protein A	5/6	ECM, CYT	Microcephaly, macrocephaly [171]
P0DPK3	*NOTCH2NLB*	Notch homolog 2 N-terminal-like protein B	4/6	ECM	Microcephaly, macrocephaly [171]
P0DPK4	*NOTCH2NLC*	Notch homolog 2 N-terminal-like protein C	5/6	ECM	Neuronal intranuclear inclusion disease (NIID) [172,173,174]; Oculopharyngodistal myopathy 3 (OPDM3) [175]
**Q9UM47**	***NOTCH3***	**Neurogenic locus notch homolog protein 3**	**14/34** [176]	CM	Cerebral arteriopathy, autosomal dominant, with subcortical infarcts and leukoencephalopathy, 1 (CADASIL1) [177,178]; Myofibromatosis, infantile 2 (IMF2) [179]; Lateral meningocele syndrome (LMNS) [180]
Q99466	*NOTCH4*	Neurogenic locus notch homolog protein 4	18/29	CM	-
Q96CW9	*NTNG2*	Netrin-G2	1/1	CM	Neurodevelopmental disorder with behavioral abnormalities, absent speech, and hypotonia (NEDBASH) [181,182]
**Q6UXH9**	***PAMR1***	**Inactive serine protease PAMR1**	**1/1** [183]	ECM	-
Q5VY43	*PEAR1*	Platelet endothelial aggregation receptor 1	3/9	CM	Cardiovascular disease [184]
**P00750**	***PLAT***	**Tissue-type plasminogen activator**	**1/1** [49]	ECM	Increased activity results in excessive bleeding; Defective release results in thrombosis or embolism [185]
**P00749**	***PLAU***	**Urokinase-type plasminogen activator**	**1/1** [47,48]	ECM	Quebec platelet disorder (QPD) [186]
P04070	*PROC*	Vitamin K-dependent protein C	1/2	ECM	Thrombophilia due to protein C deficiency, autosomal dominant (THPH3) [187]; Thrombophilia due to protein C deficiency, autosomal recessive (THPH4) [188]
P22891	*PROZ*	Vitamin K-dependent protein Z	1/2	ECM	-
P78509	*RELN*	Reelin	2/8	ECM	Lissencephaly 2 (LIS2) [189]; Epilepsy, familial temporal lobe, 7 (ETL7) [190]
Q96GP6	*SCARF2*	Scavenger receptor class F member 2	1/7	CM	Van den Ende-Gupta syndrome (VDEGS) [191]
O75093	*SLIT1*	Slit homolog 1 protein	2/9	ECM	-
O94813	*SLIT2*	Slit homolog 2 protein	3/7	ECM	-
O75094	*SLIT3*	Slit homolog 3 protein	3/9	ECM	-
Q8TER0	*SNED1*	Sushi, nidogen and EGF-like domain-containing protein 1	10/15	ECM	-
Q9NY15	*STAB1*	Stabilin-1	3/16	CM	Hyperferritinemia (HRFT) [192]
Q8WWQ8	*STAB2*	Stabilin-2	6/17	CM, CYT	-
Q6UWL2	*SUSD1*	Sushi domain-containing protein 1	2/3	CM	-
Q4LDE5	*SVEP1*	Sushi, von Willebrand factor type A, EGF and pentraxin domain-containing protein 1	4/9	ECM, CYT	Coronary artery disease [193]; artherosclerosis [194]
Q9UKZ4	*TENM1*	Teneurin-1	1/8	CM	-
Q9NT68	*TENM2*	Teneurin-2	2/8	CM	-
Q9P273	*TENM3*	Teneurin-3	1/7	CM	Microphthalmia/Coloboma 9 (MCOPCB9) [195]; Microphthalmia, syndromic, 15 (MCOPS15) [196]
Q6N022	*TENM4*	Teneurin-4	2/8	CM	Tremor, hereditary essential 5 (ETM5) [197]
P49746	*THBS3*	Thrombospondin-3	1/3	ECM	-
P35590	*TIE1*	Tyrosine-protein kinase receptor Tie-1	1/3	CM	Lymphatic malformation 11 (LMPHM11) [198]
P07911	*UMOD*	Uromodulin	3/4	ECM, CM	Tubulointerstitial kidney disease, autosomal dominant, 1 (ADTKD1) [199,200]
Q5DID0	*UMODL1*	Uromodulin-like 1	1/3	CM, CYT	-
Q6EMK4	*VASN*	Vasorin	1/1	ECM, CM	-
P13611	*VCAN*	Versican core protein	2/2	ECM	Wagner vitreoretinopathy (WGVRP) [201,202]
**Q5GFL6**	***VWA2***	**von Willebrand factor A domain-containing protein 2**	**2/2** [203]	ECM	-
Q8N2E2	*VWDE*	von Willebrand factor D and EGF domain-containing protein	3/7	ECM	-
Q9Y5W5	*WIF1*	Wnt inhibitory factor 1	2/5	ECM	-
Q0PNF2	*-*	FEX1	3/20	-	-

This list of putative POFUT1 substrates is updated from Luther et al., 2021 [59]. Proteins with experimentally confirmed *O*-fucosylation are highlighted in bold, with references provided. The number of EGF repeats containing POFUT1 consensus sequences C^2^XXXX[S/T]C^3^ out of the total EGF repeat number is listed. Any known human pathologies associated with the listed proteins (reported in UniProt) are indicated with the corresponding references. Major subcellular locations for the listed proteins (reported in UniProt) are indicated. ECM, extracellular matrix; CM, cell membrane; CYT, cytoplasm; N, nucleus; GA, Golgi apparatus.

#### 3.2.2. POFUT2 (FUT13)

The discovery of *O*-fucose on thrombospondin type 1 repeats (TSRs) occurred later than the identifying of *O*-fucose on EGF domains. Similar to EGFs, TSRs are small protein domains of 50–70 amino acids that contain six cysteines forming three disulfide bonds, albeit with distinct bonding patterns (Figure 3B). The *O*-fucose on TSRs was initially identified as glucose–fucose disaccharides on thrombospondin-1 by Hofsteenge et al. while mapping sites for *C*-mannosylation [204]. An initial consensus sequence was proposed as C^1^SX[S/T]C^2^G, which has been further refined to C^1^XX[S/T]C^2^ (group 1 TSRs) and C^2^XX[S/T]C^3^ (group 2 TSRs) through the comparison of additional TSR *O*-fucose sites identified later and site-mutagenesis studies of TSR structures [205,206,207]. Note that group 1 TSRs and group 2 TSRs have different disulfide bonding patterns (group 1: C^1^-C^5^, C^2^-C^6^, C^3^-C^4^; group 2: C^1^-C^4^, C^2^-C^5^, C^3^-C^6^), but both can be *O*-fucosylated [208,209]. Tryptophan frequently appears upstream of the consensus sequence. While it does not appear to be necessary for *O*-fucosylation, tryptophan residues intercalate with the conserved arginine residues in TSRs, forming a characteristic tryptophan-arginine ladder that forms the core of the TSR fold (Figure 3E) [210]. Tryptophan also serves as a potential site for *C*-mannosylation (consensus sequence WXXW/C) that provides further support for the folding of TSR structures [210,211]. An enzyme distinct from POFUT1 was proposed for adding *O*-fucose to TSRs, since purified, recombinant POFUT1 was unable to catalysis this reaction [46]. Recombinant *Drosophila* Ofut2, a homologue of *Drosophila* Ofut1, was shown to have the capability to *O*-fucosylate TSRs [42]. Homologues of *Ofut2* were also identified in the human (*POFUT2*), mouse (*Pofut2*), and *C. elegans* (*pofut2*) genomes.

Like POFUT1, POFUT2 is ER localized and requires a folded TSR structure for modification [46]. The fact that both POFUT1 and POFUT2 can discern folded substrate structures has led to the hypothesis that they participate in a non-canonical ER quality control process for protein folding, which will be discussed in more detail later. *Pofut2* knockout in mice is embryonic lethal [212]. These embryos die in mid-gastrulation with extensive mesoderm differentiation, suggesting that POFUT2 is a key modulator of gastrulation. Database searches with POFUT2 *O*-fucose consensus sequence in the context of a TSR identified 49 putative protein targets (splice variants are not included) (Table 2) [24,59]. Notably, most of these targets are components of the extracellular matrix, with over half belonging to the A disintegrin and metalloproteinase with thrombospondin type 1 motifs (ADAMTS) family. *Adamts9* knockout in mice leads to early embryonic lethality with phenotypes mirroring those observed in *Pofut2* knockout embryos [213]. This strongly suggests that the gastrulation defects observed in *Pofut2* knockout embryos are primarily due to the loss of *O*-fucosylation of ADAMTS9, emphasizing that *O*-fucosylation is essential for the biological function of POFUT2 protein targets. The *O*-fucose on TSR can be elongated with a glucose by β3-glucosyltransferase (B3GLCT). While no human mutations in *POFUT2* have been identified, mutations in *B3GLCT* lead to a human developmental disorder named Peters-plus syndrome (PTRPLS), which will be discussed in more detail later [214].

**Table 2:** List of putative human protein substrates of POFUT2.

UniProt ID	Gene Name	Protein Name	Consensus/Total	Subcellular Location	Known Human Pathology (If Any)
Q9UHI8	*ADAMTS1*	A disintegrin and metalloproteinase with thrombospondin motifs 1	3/3	ECM	-
O95450	*ADAMTS2*	A disintegrin and metalloproteinase with thrombospondin motifs 2	3/4	ECM	Ehlers-Danlos syndrome, dermatosparaxis type (EDSDERMS) [215]
O15072	*ADAMTS3*	A disintegrin and metalloproteinase with thrombospondin motifs 3	3/4	ECM	Hennekam lymphangiectasia-lymphedema syndrome 3 (HKLLS3) [216]
O75173	*ADAMTS4*	A disintegrin and metalloproteinase with thrombospondin motifs 4	1/1	ECM	-
**Q9UNA0**	***ADAMTS5***	**A disintegrin and metalloproteinase with thrombospondin motifs 5**	**2/2** [217]	ECM	-
**Q9UKP5**	***ADAMTS6***	**A disintegrin and metalloproteinase with thrombospondin motifs 6**	**3/5** [218]	ECM	-
Q9UKP4	*ADAMTS7*	A disintegrin and metalloproteinase with thrombospondin motifs 7	6/8	ECM	-
Q9UP79	*ADAMTS8*	A disintegrin and metalloproteinase with thrombospondin motifs 8	2/2	ECM	-
**Q9P2N4**	***ADAMTS9***	**A disintegrin and metalloproteinase with thrombospondin motifs 9**	**12/15** [219]	ECM	-
**Q9H324**	***ADAMTS10***	**A disintegrin and metalloproteinase with thrombospondin motifs 10**	**3/5** [219]	ECM	Weill–Marchesani syndrome 1 (WMS1) [220,221]
P58397	*ADAMTS12*	A disintegrin and metalloproteinase with thrombospondin motifs 12	7/8	ECM	-
**Q76LX8**	***ADAMTS13***	**A disintegrin and metalloproteinase with thrombospondin motifs 13**	**7/8** [206]	ECM	Thrombotic thrombocytopenic purpura, hereditary (TTP) [222]
Q8WXS8	*ADAMTS14*	A disintegrin and metalloproteinase with thrombospondin motifs 14	3/4	ECM	-
Q8TE58	*ADAMTS15*	A disintegrin and metalloproteinase with thrombospondin motifs 15	3/3	ECM	Arthrogryposis, distal, 12 (DA12) [223]
Q8TE57	*ADAMTS16*	A disintegrin and metalloproteinase with thrombospondin motifs 16	6/6	ECM	-
**Q8TE56**	***ADAMTS17***	**A disintegrin and metalloproteinase with thrombospondin motifs 17**	**4/5** [224]	ECM	Weill–Marchesani syndrome 4 (WMS4) [225]
Q8TE60	*ADAMTS18*	A disintegrin and metalloproteinase with thrombospondin motifs 18	4/5	ECM	Microcornea, myopic chorioretinal atrophy, and telecanthus (MMCAT) [226]
Q8TE59	*ADAMTS19*	A disintegrin and metalloproteinase with thrombospondin motifs 19	4/5	ECM	Cardiac valvular dysplasia 2 (CVDP2) [227,228]
**P59510**	***ADAMTS20***	**A disintegrin and metalloproteinase with thrombospondin motifs 20**	**12/15** [229]	ECM	-
**Q8N6G6**	***ADAMTSL1***	**ADAMTS-like protein 1**	**9/9** [207]	ECM	-
**Q86TH1**	***ADAMTSL2***	**ADAMTS-like protein 2**	**6/7** [230]	ECM	Geleophysic dysplasia 1 (GPHYSD1) [231,232]
P82987	*ADAMTSL3*	ADAMTS-like protein 3	9/10	ECM	-
Q6UY14	*ADAMTSL4*	ADAMTS-like protein 4	2/6	ECM	Ectopia lentis 2, isolated, autosomal recessive (ECTOL2) [233]; Ectopia lentis et pupillae (ECTOLP) [234]
Q6ZMM2	*ADAMTSL5*	ADAMTS-like protein 5	1/1	ECM	-
**O14514**	***ADGRB1***	**Adhesion G protein-coupled receptor B1**	**4/5** [235]	CM	-
O60241	*ADGRB2*	Adhesion G protein-coupled receptor B2	4/4	CM	-
O60242	*ADGRB3*	Adhesion G protein-coupled receptor B3	4/4	CM	-
P13671	*C6*	Complement component C6	1/3	ECM	Complement component 6 deficiency (C6D) [236]
**O00622**	***CCN1***	**CCN family member 1**	**1/1** [237]	ECM	-
**P29279**	***CCN2***	**CCN family member 2**	**1/1** [219]	ECM	-
P48745	*CCN3*	CCN family member 3	1/1	ECM, CYT	-
O95388	*CCN4*	CCN family member 4	1/1	ECM	-
O76076	*CCN5*	CCN family member 5	1/1	ECM	-
O95389	*CCN6*	Cellular communication network factor 6	1/1	ECM	Progressive pseudorheumatoid dysplasia (PPRD) [238]
**P27918**	***CFP***	**Properdin**	**4/7** [239]	ECM	Properdin deficiency (PFD) [240]
Q8IUL8	*CILP2*	Cartilage intermediate layer protein 2	1/1	ECM	-
Q96RW7	*HMCN1*	Hemicentin-1	6/6	ECM, CYT	Macular degeneration, age-related, 1 (ARMD1) [241]
B1AKI9	*ISM1*	Isthmin-1	1/1	ECM	-
O95428	*PAPLN*	Papilin	4/5	ECM	-
Q13591	*SEMA5A*	Semaphorin-5A	2/7	CM	-
Q9P283	*SEMA5B*	Semaphorin-5B	2/5	CM	-
**Q9HCB6**	***SPON1***	**Spondin-1**	**5/6** [242]	ECM	-
**A2VEC9**	***SSPOP***	**SCO-spondin**	**16/25** [243]	ECM	-
**P07996**	***THBS1***	**Thrombospondin-1**	**3/3** [204]	ECM	-
**P35442**	***THBS2***	**Thrombospondin-2**	**3/3** [209]	ECM	Intervertebral disc disease (IDD) [244]
Q9NS62	*THSD1*	Thrombospondin type-1 domain-containing protein 1	1/1	ECM	Lymphatic malformation 13 (LMPHM13) [245,246]; Aneurysm, intracranial berry, 12 (ANIB12) [247,248]
Q6ZMP0	*THSD4*	Thrombospondin type-1 domain-containing protein 4	3/6	ECM	Aortic aneurysm, familial thoracic 12 (AAT12) [249]
Q9UPZ6	*THSD7A*	Thrombospondin type-1 domain-containing protein 7A	7/19	ECM, CM	Pathogenic autoantigen in membranous nephropathy (MN) [250,251]
Q9C0I4	*THSD7B*	Thrombospondin type-1 domain-containing protein 7B	6/18	CM	-

This list of putative POFUT2 substrates is updated from Luther et al., 2021 [59]. Proteins with experimentally confirmed *O*-fucosylation are highlighted in bold, with references provided. The number of TSRs containing POFUT2 consensus sequences C^1−2^XX[S/T]C^2−3^ out of the total TSR number is listed. Any known human pathologies associated with the listed proteins (reported in UniProt) are indicated with the corresponding references. Major subcellular locations for the listed proteins (reported in UniProt) are indicated. ECM, extracellular matrix; CM, cell membrane; CYT, cytoplasm.

#### 3.2.3. POFUT3 (FUT10) and POFUT4 (FUT11)

In 2002, Roos et al. used a whole-genome approach to search for enzymes that involved fucosylated glycan metabolism in *Drosophila* genome [252]. A *Drosophila* fucosyltransferase, *FucTB*, was among their findings. Using the *FucTB* sequence to search the human genome, two human orthologs were identified. These human genes were named *FUT10* and *FUT11*, as they were identified after *FUT9*. Due to the presence of the two conserved motifs shared with other human α3-FUTs (FUT3–7 and FUT9), it was proposed that they were responsible for GDP-fucose donor binding, and FUT10 and FUT11 were initially presumed to be α3-FUTs.

The *Drosophila FucTB* gene was cloned together with *FucTA* and *FucTC* as homologs in 2001, but only FucTA demonstrated clear activity as a core α3-FUT for *N*-glycans [253]. No activity was detected for FucTB in generating either core-fucosylated *N*-glycans or Lewis-type glycans [253,254]. Efforts to experimentally demonstrate fucosyltransferase activity using free glycan substrates for the mouse homologs, Fuc-TX and Fuc-TXI, and the honeybee FucTB homolog were also unsuccessful, although Fuc-TXI was found to possess GDP-fucose hydrolase activity [255,256,257]. Studies exploring the fucosyltransferase activity of human FUT10 and FUT11 have yielded varied results. Mollicone et al. reported core α3-FUT activity for both FUT10 and FUT11, showing they modify the innermost GlcNAc moiety of the chitobiose unit on *N*-glycans via an α3-linkage using synthetic glycan substrates [258]. In contrast, Kumar et al. reported that FUT10 is involved in an α3-FUT activity for the synthesis of Lewis X (Le^X^) epitopes in neuroblastoma cells [259]. It is important to note that neither of these studies used purified enzymes to detect enzymatic activity; instead, crude lysates from FUT10/11-transfected cells were used as the enzyme source. This raises the possibility that the activity observed in their studies could be due to indirect effects from protein substrates modified by FUT10/11 or from other endogenous FUTs in the cell lysate. The substrate specificity of FUT10 and FUT11 remained unclear for a decade until recently (Table 3).

In 2024 a new type of domain-specific *O*-fucosylation was identified in a proteomic study on human platelet releasate [163]. The fucose was added at a high stoichiometry on the elastin microfibril interface (EMI) domain of Multimerin-1 (MMRN1), a key site for protein–protein interaction (Figure 4C). EMI is a cysteine-rich domain with three conserved disulfide bonds, like the EGF or TSR domains but larger (Figure 3C). Neither POFUT1 nor POFUT2 was found to be responsible for this modification, suggesting the existence of a previously unidentified POFUT specific for the EMI *O*-fucosylation [45]. AlphaFold2 was employed to screen potential binding interactions between human FUTs and the EMI domain [45]. FUT10 and FUT11 demonstrated significant EMI binding that was not observed with other FUTs. The AlphaFold2 predictions were further validated through co-immunoprecipitation assays, confirming the interaction between FUT10/11 and EMI domains in a cellular context. In vitro enzymatic assays with purified, recombinant FUT10 and FUT11 revealed robust POFUT activity with EMI substrates. Furthermore, knocking out either *FUT10* or *FUT11* in HEK293T cells significantly reduced EMI *O*-fucosylation, while knocking out of both enzymes completely abolished the modification. These findings imply that FUT10 and FUT11 function as novel POFUTs for EMI domains, leading to their renaming as POFUT3 and POFUT4, respectively.

FUT10 and FUT11 have been annotated as α3-FUTs (see Q6P4F1 and Q495W5 in UniProt). They were classified to the CAZy GT10 family, along with the invertebrate and plant core α3-FUTs and all terminal α3/4-FUTs [27]. However, multiple clues suggest that they are in a distinct family that should be separated from other α3-FUTs. The α3/4-FUT family shares five conserved peptide motifs: motifs I–III contribute to acceptor substrate recognition, while motifs IV and V contribute to recognition and binding of the donor substrate GDP-fucose [258,267,268]. FUT10 and FUT11 show high sequence homology with other human GT10 family members (FUT3–7 and FUT9) in motifs IV and V, with strong conservation of residues essential for enzymatic activity and GDP-fucose binding [258]. This homology is also reflected in the 3D structural alignment of AlphaFold2 predicted structures of FUT10/11 with crystal structures of *H. pylori* FucT and human FUT9, where the C-terminal Rossmann-like domain that responsible for GDP-fucose binding shows a high degree of similarity [45]. In contrast to motifs IV and V, FUT10 and FUT11 show limited homology with other α3-FUTs in motifs I–III, both in primary peptide sequence and 3D structural alignment [45,252,256,258]. All α3/4-FUTs possess the conserved peptide sequence [F/I/V]HH[R/W][E/D] in motif II, a characteristic motif for lactosamine acceptor binding, as a single amino acid change (W to R) can convert the α4 activity of FUT3 to α3 activity [269]. Instead, FUT10 and FUT11 present a different conserved peptide motif FYGTDF at the equivalent position, indicating they do not use lactosamine as acceptors. Additionally, FUT10 and FUT11 contain a unique C-terminal extension predicted to generate additional contacts with EMI substrates for aiding in substrate selection [45]. These differences suggest that FUT10 and FUT11 have a distinct acceptor substrate compared to other α3/4-FUTs family members. Phylogenetic analysis indicates that FUT10 and FUT11 belong to an evolutionary distinct group compared to other α3/4-FUTs [252,258]. These enzymes are ancient, originating at about 830 MYA (Million Years Ago), and possess a split polyexonic genomic structure, which is clearly distinct from the monoexonic α3/4-FUTs that originated around 450 MYA. Note that POFUT1 and POFUT2 are also ancient enzymes (originated >1000 MYA) with a polyexonic structure [270].

Like POFUT1 and POFUT2, POFUT3 (FUT10) and POFUT4 (FUT11) are ER-localized enzymes [45]. They also require folded domain structures for efficient modification and participate in a non-canonical ER quality control pathway for protein secretion. In addition to these similarities, POFUT3/4-mediated EMI *O*-fucosylation displays several unique characteristics. First, unlike EGF repeats and TSRs which are often embedded within proteins as tandem repeats, the EMI domain is consistently singular and located at the N-terminus of proteins. Second, multiple fucose residues can be added to the EMI domain in a spatially adjacent manner. For instance, the EMI domain on MMRN1 can hold two fucose residues, while the EMI domain on MMRN2 can hold three [45]. These fucose residues on EMI domains are exclusively observed as monosaccharides, whereas those on EGF repeats and TSRs are frequently elongated. Screening more EMI-containing proteins for *O*-fucosylation would help decipher the code for fucose addition and refine the POFUT3/4 consensus sequence. There are 17 EMI-containing human proteins annotated in UniProt that are potential substrates for POFUT3/4 (Table 3). However, given the different subsets of EMI domain structures, it is possible only some are *O*-fucosylated [271,272,273].

No human genetic diseases have been identified with mutations in *POFUT3* or *POFUT4*. However, aberrant expression of POFUT4 has been associated with several types of cancer [274,275,276,277,278,279,280]. In mice, POFUT3 and POFUT4 are expressed ubiquitously in embryos throughout development but show distinct tissue-specific expression patterns in adults [255,256,258]. Notably, they are strongly expressed in Purkinje cells in the brain, suggesting a potential role for these enzymes and their substrates in motor coordination [255]. POFUT3 is required for the maintenance of mouse embryonic stem (ES) cells and neural stem cells (NSCs) [259]. Overexpression of POFUT3 enhanced the self-renewal of NSCs, while knocking down *POFUT3* induced the differentiation of NSCs and ES cells. Knocking down *POFUT4* in zebrafish embryos resulted in malformations of the posterior trunk and tail, indicating its important roles in vertebrate development [256]. The POFUT3/4 protein substrates and potential mechanisms involved in these events remain unknown. Generating *POFUT3* and/or *POFUT4* knockout mice would provide deeper insights into their biological functions in embryonic development, neurological function, and stem cell maintenance.

## 4. *O*-Fucosylation: Physiological and Pathological Significance

### 4.1. Regulation of Lymphoid and Myeloid Cell Development by Notch O-Fucosylation

As previously mentioned, Notch is a key regulator in various developmental processes, frequently influencing cell fate decisions at multiple stages as cells differentiate toward specific cell types. In hematopoiesis, proper Notch signaling is essential for the survival, proliferation, and cell fate determination at various stages of lineage commitment, including myeloid cell production in the bone marrow, T cells development in the thymus, and marginal zone (MZ) B cells development in the spleen. *O*-fucose glycans incorporated to Notch extracellular domain by POFUT1 and further elongated by Fringe modulate the strength of Notch signaling, thus playing critical roles in these Notch-regulated programs (Figure 4A).

Throughout adult life, blood cell production is maintained by a group of pluripotent hematopoietic stem cells (HSCs) located in the bone marrow. HSCs generate multipotent progenitor populations (MPPs) with increased lineage-specific potentials. Within this MPP pool, lymphoid-primed MPPs (LMPP or MPP4) subsequently give rise to common lymphoid progenitors (CLPs) and common myeloid progenitors (CMPs), which are committed to the lymphoid lineage (T, B, and natural killer cells) and the myeloid lineage (neutrophils, monocytes, erythrocytes, and more), respectively [281]. Notch signaling influences this decision by favoring a lymphoid over myeloid lineage outcome [282,283]. Conditional knockout *Pofut1* in mice HSCs within the bone marrow leads to reduced T lymphopoiesis in the thymus and reduced marginal-zone B cells production in the spleen, whereas myeloid cells in bone marrow were increased [284]. Restoration of Notch1 signaling rescued these phenotypes. The *Pofut1* knockout in HSCs completely abrogated the binding of cell surface Notch with Delta ligands and suppressed the activation of downstream Notch target genes. In another study, the selective deletion of the Notch ligand *DLL4* in bone-producing osteocalcin (Ocn)-expressing cells, which highly express DLL4 in the bone marrow, results in a decrease in intrathymic T cell precursors and mature T cells production [285]. These findings suggest that Notch *O*-fucosylation is essential for maintaining hematopoietic lineage homeostasis by promoting lymphoid development and suppressing overt myelopoiesis. Interestingly, pan-Notch inhibition through the conditional deletion of *RBP-Jκ* led to more severe phenotypes than the deletion of *Pofut1* in HSCs, suggesting other *O*-glycans on Notch may contribute to this Notch-regulated process [285]. The roles of other Notch *O*-glycans in hematopoiesis were recently reviewed in detail [65].

T cell maturation takes place in the thymus, where lymphocyte precursors released from the bone marrow migrate through blood vessels and colonize the thymus as thymus-seeding progenitors (TSPs). Upon entering the thymus, these cells initiate a differentiation process that includes crucial events such as T cell lineage commitment and the choice between αβ and γδ T-cell lineages, as they migrate from the corticomedullary junction towards the outer cortical zone. During this migration, the progenitor cells traverse a network of thymic stromal cells that express Notch ligands, initiating Notch signaling in the progenitor cells and guiding them through the differentiation programs. This requirement of Notch signaling in T cell development is well-documented. TSPs possess robust potential to generate non-T cell lineages, including B cells, myeloid cells, and dendritic cells (DC) [286]. NOTCH1-DLL4 signaling compels these cells to commit to a T cell fate while simultaneously suppressing their potential to adopt other lineages. Disrupting Notch signaling in HSCs through conditional deletion of *Notch1* or *RBPJκ* blocks early-stage T cell development, causing T cell progenitors to differentiate into B cells and myeloid cells in the thymus [287,288,289,290]. By using fucose analogs (6-alkynyl fucose and 6-alkenyl fucose) that incorporate into Notch and specifically inhibit Delta-induced Notch signaling in an in vitro OP9 co-culture system, the differentiation of bone marrow stem cells into T cell progenitors was completely abolished, indicating the important roles of *O*-fucose in Notch-regulated T cell lineage commitment [291]. Interestingly, overexpression of Lunatic Fringe (Lfng) in the thymus, which enhances Notch binding with Delta ligands, unexpectedly inhibits Notch activation in TSPs [289]. This is because when Lfng is overexpressed in CD4/CD8 double-negative (DN) 3 and CD4/CD8 double-positive (DP) cells, it transforms them into ‘super-competitors’ for the limited intrathymic niches supplying Delta ligands [292]. This limits the access of early T-cell progenitors towards these ligands, which are needed for the required Notch signaling for their differentiation.

NOTCH2-DLL1 signaling is required for the development of marginal zone (MZ) B cells in the spleen [293,294]. Conditional deletion of *Pofut1* in HSCs reduced marginal-zone B cells production in spleen [284]. Although neither Lfng nor Manic Fringe (Mfng) is indispensable for MZ B cell development, the loss of both Fringes significantly impairs the production of MZ B cells in spleen [295]. Therefore, Lfng and Mfng cooperatively enhance the NOTCH2-DLL1 signaling to promote the development of MZ B cells.

### 4.2. Cancer with Altered O-Fucosylation

Notch signaling plays diverse roles during development and tissue homeostasis. A growing number of cancers are linked to dysregulated Notch signaling, where Notch can act as either an oncogene or a tumor suppressor, depending on the cellular context [296]. As a crucial regulator of Notch signaling, altered *O*-fucosylation is commonly observed in cancer. Here, we highlight prototypical cancers where altered *O*-fucosylation plays a role in their pathological mechanisms. This alteration can involve changes in POFUT1 levels or activity, or mutations in Notch that impact its *O*-fucosylation.

POFUT1 is localized in the chromosomal region 20q11.21, which is frequently amplified in colorectal cancer (CRC) [297]. This copy number variations (CNVs) in CRC results in elevated POFUT1 expression in tumors compared to non-tumor adjacent tissues [298]. The high expression of POFUT1 in CRC from stage I is associated with the metastatic process. ShRNA-mediated knockdown of POFUT1 downregulates NOTCH1 signaling, leading to decreased cell proliferation, migration, and induced apoptosis in CRC cells in vitro, as well as suppression of CRC tumor growth and transplantation in vivo [299]. In less frequent CRC cases with no chromosomal amplification and POFUT1 overexpression, gain-of-function mutations in POFUT1 have been identified, potentially contributing to tumor progression [299]. POFUT1 is overexpressed in hepatocellular carcinoma (HCC), and its high expression correlates with poor prognosis in HCC patients [300,301]. Elevated POFUT1 levels accelerated the proliferation and migration of HCC cells and increased their binding to DLL1, consequently enhancing Notch signaling activity. Overexpression of POFUT1 is also observed in glioblastoma [302,303], gastric cancer [304], oral cancer [305], and breast cancer [306], correlating with increased Notch activity and aggressive phenotypes, such as increased cell proliferation, invasion, metastasis, or larger tumor size. Interestingly, in cancer types where Notch functions as a tumor suppressor, such as in muscle-invasive bladder cancer (MIBC), low levels of POFUT1 mRNA are associated with poor survival in MIBC patients after radical cystectomy [307,308]. These findings highlight the potential of POFUT1 as a diagnostic marker and therapeutic target for these cancers.

In anaplastic large cell lymphoma (ALCL), the Notch pathway ranks among the pathways that are most enriched in mutations through gene-set enrichment analysis. NOTCH1 p.T311P (eliminates *O*-fucose site on EGF8) and p.T349P (eliminates *O*-fucose site on EGF9) missense mutations were found in patients’ tumor samples by whole-exome sequencing [309]. Interestingly, overexpression of these mutations in HEK293T cells led to increased proliferation compared to cells with wild-type NOTCH1. While the detailed mechanism remains unclear, this suggests a correlation between alterations in specific *O*-fucose sites on NOTCH1 and cancer. Recently, by using glycoproteomic site mapping and cell-based Notch assays, nine cancer-associated Notch variants were investigated to determine their impact on Notch *O*-fucosylation, ligand binding and signaling activity [310]. Two variants led to a gain of function in NOTCH1 (G309R and N386T), six led to a loss of function (G310R, T311P, G347S, T349P, D464N, and A465T), and one had minimal effects (G230R). This suggests that point mutations in Notch can alter its *O*-fucosylation, consequently affecting Notch signaling activity and being linked to cancer-related processes.

Elevated expression of POFUT4 was observed in gastric cancer (GC) tissues compared to non-tumor adjacent tissues and is correlated with poor prognosis and survival in GC patients [274,276]. Knockdown of *POFUT4* reduced the proliferation and migration of GC cells by suppressing the PI3K/AKT signaling pathway. Overexpression of POFUT4 and its correlation with poor prognosis were also reported in multiple other cancer types, including clear cell renal cell carcinoma (ccRCC) [279,280], colon adenocarcinoma (COAD) [278], hepatocellular carcinoma (HCC) [277], and pancreatic cancer (PC) [275]. Although the related protein substrates and mechanisms underlying POFUT4’s role in cancer development and progression remain unknown, these findings suggest that targeting POFUT4 holds therapeutic potential and may serve as a novel biomarker for cancer prognosis.

### 4.3. Peters-Plus Syndrome (PTRPLS)

Failure to add glucose to *O*-fucose on TSRs causes human Peters-plus syndrome (PTRPLS, OMIM #261540), an autosomal recessive congenital disorder of glycosylation (CDG) characterized by Peters’ anomaly of the eye (anterior eye chamber segment dysgenesis), craniofacial defects (widened forehead and cleft lip/palate), short stature, brachydactyly, and developmental delay [214]. PTRPLS is caused by loss-of-function mutations in β3-glucosyltransferase (B3GLCT), an enzyme responsible for extending *O*-fucose monosaccharide to the Glucoseβ1-3Fucose disaccharides on TSRs (Figure 3B) [311,312,313,314]. PTRPLS-like patients only share a subset of phenotypes observed in PTRPLS patients, and some of them also have missense mutations in B3GLCT [311]. In contrast to PTRPLS mutations that lead to a complete loss of enzymatic activity, PTRPLS-like mutations retained enzymatic activity with only minor destabilizing effects [315]. This partially explains the milder phenotypes observed in PTRPLS-like patients.

Over half of the putative POFUT2 targets are members of the ADAMTS/ADAMTS-like family, suggesting that these proteins could be the primary biological targets for B3GLCT (Table 2 and Figure 4B). These secreted ADAMTS proteases and non-catalytic members of the family (ADAMTS-like proteins) are important components of the extracellular matrix (ECM), and are implicated in diverse biological events, including embryogenesis and angiogenesis [316,317]. Numerous congenital disorders caused by mutations in *ADAMTS/ADAMTS*-like genes display overlapping phenotypes with PTRPLS patients. For instance, mutations in *ADAMTSL2* cause human Geleophysic dysplasia 1 (GPHYSD1, OMIM #231050), where patients exhibit overlapped phenotypes such as short stature and short hands and feet [230,232]. Mutations in *ADAMTS10* cause Weill–Marchesani syndrome 1 (WMS, OMIM #277600), with overlapped phenotypes including short stature, brachydactyly and eye anomalies [220]. These suggest that impaired *O*-fucosylation of the ADAMTS/ADAMTS-like family proteins is likely the primary biological mechanism responsible for the phenotypes observed in PTRPLS patients.

POFUT2 and B3GLCT are demonstrated to mediate a noncanonical ER quality-control process for the secretion of TSR-containing proteins (molecular details will be discussed later) [318]. POFUT2 is required for the secretion of almost all ADAMTS/ADAMTS-like proteins tested to date (ADAMTS6 [218], ADAMTS9 [219,229], ADAMTS10 [218], ADAMTS13 [318], ADAMTS17 [224], ADAMTS20 [229], ADAMTSL1 [318], ADAMTSL2 [318]), whereas the impact of B3GLCT on secretion varied among proteins. For instance, secretion of ADAMTS20, ADAMTSL1, and ADAMTSL2 is profoundly reduced with *B3GLCT* knockdown/out [229,318]. In contrast, ADAMTS9 and ADAMTS13 secretion is moderately reduced by 20%, and ADAMTS17 secretion is unaffected [219,224,229,318]. Considering the early embryo lethality observed in *Pofut2*-null mice and the less severe phenotypes in PTRPLS patients, it implies that the pathology of PTRPLS is due to defects in a specific subset of POFUT2 targets that are sensitive to the loss of extended glucose.

### 4.4. Spondylocostal Dysostosis 3 (SCDO3)

Failure to add GlcNAc to *O*-fucose on NOTCH1 EGF repeats causes Spondylocostal Dysostosis 3 (SCDO3, OMIM #609813), an autosomal recessive disorder in human that characterized by multiple defects in vertebral segmentation, including hemivertebrae, butterfly vertebrae, fusion or missing vertebrae, scoliosis, and rib anomalies [319]. SCDO3 is caused by loss-of-function mutations in *Lunatic Fringe (Lfng)*, leading to mis-localization, reduced stability or impaired enzymatic activity of proteins [320]. More than twenty *Lfng* variants have been identified in SCDO3 patients, with the mutational spectrum evenly distributed across all exons [321]. Fringe modifications play a crucial role in Notch signaling by fine-tuning Notch-ligand interaction, enhancing signaling from Delta-like ligands and inhibiting signaling from Jagged ligands. Delta-like 1:NOTCH1 signaling is central to somite formation during embryonic development [322,323]. *Lfng* knockout in mice results in multiple somitogenesis defects similar to those reported in SCDO3 patients and causes perinatal lethality due to malformed rib cages [324,325]. In contrast, *Rfng* or *Mfng* knockouts in mice exhibit no obvious phenotypes, while triple knockouts of all three *Fringes* show the same phenotypes as *Lfng* knockout mice [326,327,328].

### 4.5. Other Biological Processes Where O-Fucosylation May Play a Role

In addition to the biological events discussed above, *O*-fucose glycans have been identified in numerous other biologically important proteins, where they play crucial roles in modulating protein function. POFUT1-mediated *O*-fucosylation of Agrin, a key regulator of postsynaptic differentiation at the neuromuscular junction (NMJ), has been demonstrated to determine its acetylcholine receptor clustering activity [81]. The *O*-fucose moiety on TSR3 of BAI1 (ADGRB1), added by POFUT2, directly interacts with the RTN4 receptor, enabling high-affinity interactions that regulate neuronal development (Figure 5C) [235]. Mutating the POFUT3/4 *O*-fucose sites within the EMI domain of MMRN1, an essential platelet component supporting platelet adhesion and thrombus formation, significantly reduces its secretion. While mutating the POFUT1 *O*-fucose site within the EGF domain leads to a complete loss of MMRN1 secretion, implying an as-yet-unidentified role for *O*-fucosylation in platelet functions [163]. We envision that a deeper profiling of the *O*-fucose proteome and investigation into the mechanisms by which *O*-fucose affects protein functionality will uncover more biological significance of *O*-fucosylation in the future.

## 5. Molecular Mechanisms of How *O*-Fucose Regulates Protein Functions

### 5.1. O-Fucosylation Forms Direct Contacts with Binding Partners

Out of the 36 EGF repeats in the extracellular domain of mouse NOTCH1, 20 contain the *O*-fucose consensus sequence, and 17 have been confirmed to be modified at high stoichiometries (Figure 4A) [67]. Similar results were observed in *Drosophila* NOTCH and mouse NOTCH2 [66,329]. These heavily decorated *O*-fucose glycans are required for the proper function of Notch. Through the examination of individual *O*-fucose effects on NOTCH1 activity via site-directed mutagenesis, it was found that *O*-fucose on EGF8 and EGF12 had the most significant impact on the binding of NOTCH1 with DLL1 and JAG1 ligands [67]. Similar results were obtained with NOTCH2 [66]. In vitro assays using purified human NOTCH1 EGF11–13 also demonstrated that the addition of *O*-fucose enhanced its binding to DLL1 and JAG1 ligands [74].

Evidence confirming the direct interaction of *O*-fucose on NOTCH1 EGF8 and EGF12 with ligands is provided by two co-crystal structures: one between a portion of NOTCH1 ligand-binding domain (EGF11–13) and a portion of DLL4 [72], and the other between the NOTCH1 ligand-binding domain (EGF8–12) and a portion of JAG1 (Figure 5A,B) [73]. Both co-crystal structures showed direct contacts between *O*-fucose on EGF12 with the backbone and side-chain residues of both ligands. The NOTCH1-JAG1 structure also showed a direct contact of *O*-fucose on EGF8 with JAG1. Unlike many other glycan modifications, *O*-fucose residues display thermal mobility comparable to amino acids, acting as ‘surrogate amino acids’ to form specific and essential contacts with residues on binding partners, thereby facilitating ligand binding. This direct intermolecular interaction generated by *O*-fucose glycans in protein–protein interactions was also observed in the co-crystal structure of BAI1 with RTN4 receptor (Figure 5C) [235]. The Glucoseβ1-3Fucose disaccharide of the BAI1 TSR3 domain directly interacts with the RTN4 receptor, enabling high-affinity binding and regulating neuronal development.

### 5.2. O-Fucosylation Generates Intramolecular Interactions That Stabilize Protein Domains and Facilitate Secretion

The appropriate pairing of cysteines to form correct disulfide bonds is a crucial step during protein folding in the ER, and this presents a particular challenge for proteins that contain multiple, tandem cysteine-rich domains such as EGFs and TSRs. It provides a strategic rationale for cells to evolve dedicated quality-control mechanisms to meet the specialized requirements for the folding of these domains. As mentioned above, POFUT1–4 are localized in the ER and only modify properly folded domain structures. These led to the hypothesis that the ER-localized POFUTs participate in a non-canonical ER quality control system for the folding and secretion of proteins containing cysteine-rich domains.

**Figure 6:**
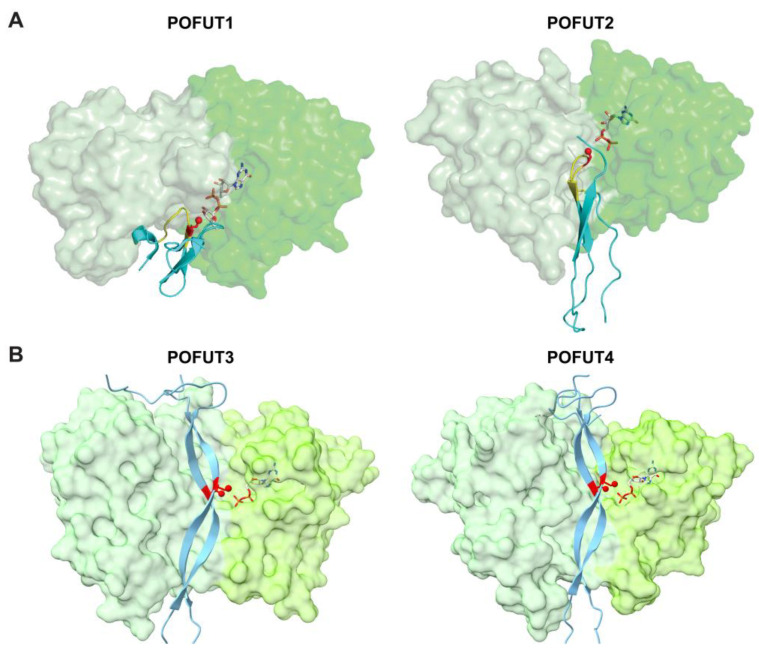
The cleft in each POFUT shows a high degree of complementarity with its respective substrate domains. (**A**) Crystal structures of POFUT1 with EGF domain (left panel, overlay of PDB IDs 5KXH and 5KY3) and POFUT2 with TSR (right panel, PDB ID 5FOE). (**B**) AlphaFold2-multimer predicted structure of human POFUT3 (FUT10) (left panel, residues 81–479) and human POFUT4 (FUT11) (right panel, residues 73–492) with human MMRN1 EMI domain. POFUT1, POFUT2, POFUT3 and POFUT4 enzymes are displayed in surface representations with A domains in light green and B domains in darker green. Folded domains (EGF repeats for POFUT1; TSR for POFUT2; EMI for POFUT3 and POFUT4) are in cyan cartoon with respective consensus sequences of POFUT1 and POFUT2 in yellow. Acceptor amino acid is shown in red sticks, with the acceptor oxygen as a red sphere. Donor nucleotides, white sticks. Panel A is modified with permission from Ref. [332].

In co-crystal structures of mouse POFUT1 with EGF domains from Notch, the binding interface between POFUT1 and EGF showed a high degree of complementarity, with approximately one-third of the EGF’s surface area being deeply embedded within the groove of POFUT1 (Figure 6A) [330]. The EGF region that contains *O*-fucose consensus motif C^2^XXXX[S/T]C^3^ is buried at the bottom and the hydroxyl group of serine or threonine to be modified is precisely oriented to the position necessary for the fucose transfer. Similarly, in co-crystal structures of *Caenorhabditis elegans* POFUT2 (CePOFUT2) with human TSR1, nearly half of the TSR1 surface is embedded within the groove of CePOFUT2 (Figure 6A) [205]. Likewise, the AlphaFold2-multimer predicted structures of POFUT3/4 and the MMRN1 EMI domain revealed a highly confident interface complementarity, with fucose sites positioned in close proximity to the putative GDP-binding pocket (Figure 6B) [45]. These ‘hand-in-glove’ complementary interactions between enzymes and their respective protein domain structures elegantly explain why the enzyme necessitates a folded structure and a specific consensus sequence for modification. This suggests that the POFUTs are folding sensors for their respective domains.

Glycan modifications are usually too flexible to be observed in crystal structures. However, *O*-fucose glycans are remarkably visible. Their thermal stability (based on B factors) is comparable to that of the underlying amino acids due to numerous intramolecular interactions [72,74]. In the crystal structures of human NOTCH1 EGF11–13 modified with GlcNAcβ1-3Fucose disaccharide, the glycan residues lay down on the surface of EGF12, forming multiple intramolecular contacts with neighboring amino acid residues (Figure 3D) [74]. Through a combination of MD simulations, X-ray crystallography, and NMR, the *O*-fucose glycans on TSR3 of THBS1 were shown to make intramolecular interactions with adjacent amino acids resulting in shielding of the C2-C6 disulfide bond to protect it from reduction [331]. Such interactions were also identified by MolProbity in several other TSR-containing proteins, such as ADAMTS13 and Properdin (Figure 3E) [44,79]. For the EMI domain, although no crystal structures are available, the AlphaFold2-predicted structure of the MMRN1 EMI domain shows that the two fucose sites are spatially adjacent but reside on two separate, antiparallel β-strands (Figure 3C) [45]. It is possible that the fucose residues generate intramolecular interactions with spatially adjacent amino acid residues on the other β-strand, bringing the two β-strands closer together, thereby restricting their flexibility and compacting the EMI structure.

The addition of an *O*-fucose to EGF or TSR domain stabilizes the folded structures and protects them from unexpected unfolding due to environmental factors. *O*-fucosylated EGF repeats and TSRs showed a significantly reduced unfolding rate in reductive unfolding assays compared to their unmodified counterparts [318,333]. Additionally, the refolding of unmodified TSRs was accelerated in the presence of POFUT2 and GDP-fucose in vitro, whereas the addition of POFUT2 alone did not have the same effect [318]. This implies that the transferred *O*-fucose, rather than POFUT2, contributes to the acceleration of the folding rate. This stabilizing effect generated by the *O*-fucose glycans drives the domain into an energy well, preventing the domains from re-entry into the folding cycle, and consequently facilitating protein folding and secretion.

In line with this, several studies have shown that disruptions in *O*-fucosylation lead to defects in protein secretion. As mentioned above, knockdown or knockout of *POFUT2* in cells eliminates the secretion of almost all ADAMTS/ADAMTS-like proteins tested to date in HEK293T cells. Mutating *O*-fucose sites within the EMI domain or knockout of both *POFUT3* and *POFUT4* resulted in a substantial reduction in the secretion of EMI-containing proteins [45,163]. Knockdown of *Drosophila Ofut1* led to reduced cell surface expression of NOTCH and its accumulation in the ER [334]. Interestingly, this secretion defect can be partially rescued by an enzymatically inactive form of POFUT1 (R240A mutant), suggesting that Ofut1 might possess chaperone activity independent of its enzymatic function. However, mice carrying POFUT1 with point mutations at the equivalent position exhibited severe defects in somite formation, resembling those observed in *Pofut1* null mice [335]. Additionally, the POFUT1 R240A mutant showed significantly reduced effectiveness in rescuing Notch signaling in *POFUT1* knockout U2OS cells when compared to the wildtype POFUT1 [336]. The chaperone activity of POFUT1 may depend on the specific context within different organisms and can yield distinct biological functions. *POFUT1* knockout in different cellular contexts led to varying outcomes in reducing cell surface Notch levels. For instance, *POFUT1* knockout in HEK293T cells resulted in ~60% reduced surface expression of NOTCH1, whereas *Pofut1* knockout in mouse embryonic stem cells displayed a similar level of cell surface NOTCH compared to the wild type [333,337]. The variation observed may stem from the distinct regulation of other chaperones that compensate for the folding and secretion defects in Notch within different cellular contexts.

## 6. Inhibitors/Modulators of *O*-Fucosylation

To date, no specific inhibitors for POFUT1, POFUT2, POFUT3, or POFUT4 have been identified. As discussed above, POFUTs play crucial roles in diseases, including various types of cancer. Having specific inhibitors or modulators for these enzymes would hold significant therapeutic potential and serve as valuable research tools. A virtual compound library screening with available POFUT structures could serve as a starting point for identifying potential inhibitors.

While not directly targeting POFUTs, specific fucose analogs like 5-thio-fucose (5T-Fuc) [338], 2-deoxy-2-fluoro-fucose (2F-Fuc) [339], 6-deoxy-6-fluoro-fucose (6F-Fuc) [340], 6,6,6-trifluoro-fucose (Fucostatin) [341], 6-alkynyl-fucose (6-AF) [342], 7-alkynyl-fucose (7-AF) [343], and β-carbafucose [344] are pan-inhibitors of cellular fucosylation. These compounds either antagonizing enzymes of the de novo GDP-fucose biosynthetic pathway or destabilizing the oxocarbenium ion-like transition states during the transfer reaction. Furthermore, 6-AF can enter the salvage pathway and be converted into GDP-6-AF, which is well-tolerated by POFUT1 and POFUT2 for incorporation into their respective protein targets [291,345,346]. The incorporated 6-AF can alter the functions of proteins, as exemplified in the case of Notch, where the incorporation of 6-AF selectively inhibits DLL ligands-mediated Notch signaling but not JAG ligands [291]. 6-AF inhibits most other FUTs, including POFUT3 and 4 [45,346]. Therefore, fucose analogs could serve as valuable tools for *O*-fucosylation research and hold therapeutic potential for their capacity to modulate the functions of proteins.

## 7. Conclusions

Given the biological importance and therapeutic potential of *O*-fucosylation, identifying proteins that are modified with *O*-fucose and understanding its role in modulating protein function would be of great value. Notable progress has been made in the last decade, including site-mapping of more EGF/TSR-containing proteins, successful crystallization of key POFUT target proteins (such as NOTCH1 and BAI1) and several POFUTs, deeper insights into the molecular mechanisms by which *O*-fucose glycans regulate protein function, and the discovery of a novel domain-specific *O*-fucosylation mediated by two uncharacterized POFUTs. Despite these remarkable progresses, much work remains to be done. First, while extensive research has focused on prominent *O*-fucosylated proteins such as Notch and the ADAMTS family, investigation into other proteins is needed. The *O*-fucosylation status of many putative POFUT substrates remains unconfirmed, yet they may have important biological functions. A comprehensive profiling of the *O*-fucose proteome is necessary. Second, the discovery of EMI *O*-fucosylation by POFUT3/4 has opened a new avenue for research, with much remaining to be explored in this emerging field. Generating *POFUT3* and/or *POFUT4* knockout mice and tissue-specific conditional knockout mice would be valuable for studying POFUT3/4-related diseases. Third, we are just beginning to understand the molecular mechanisms of how *O*-fucose modulates protein functions. While the role of *O*-fucose on NOTCH1 EGF8 and EGF12 in ligand binding is well-studied, fucose residues on other EGF repeats are also necessary for proper Notch function and may have distinct regulatory mechanisms. Additionally, little is known about how the *O*-fucose on TSRs and EMIs affects protein intermolecular interactions, while TSRs and EMIs are frequently localized within the regions involved in protein–protein interactions. Lastly, specific POFUT inhibitors are still lacking but would greatly benefit both research and therapeutic drug development. We envision that efforts to answer these questions will deepen our understanding of *O*-fucosylation and further advance its application in disease treatment.

## Figures and Tables

**Table 3:** List of putative human protein substrates of POFUT3 and POFUT4.

UniProt ID	Gene Name	Protein Name	Subcellular Location	Known Human Pathology (If Any)
Q96A83	*COL26A1*	Collagen alpha-1(XXVI) chain	ECM	-
Q9UHF1	*EGFL7*	Epidermal growth factor-like protein 7	ECM	-
Q99944	*EGFL8*	Epidermal growth factor-like protein 8	ECM	-
**Q96A84**	***EMID1***	**EMI domain-containing protein 1** [45]	ECM	-
Q9Y6C2	*EMILIN1*	EMILIN-1	ECM	Neuronopathy, distal hereditary motor, autosomal dominant 10 (HMND10) [260,261]
Q9BXX0	*EMILIN2*	EMILIN-2	ECM	-
Q9NT22	*EMILIN3*	EMILIN-3	ECM	-
O75095	*MEGF6*	Multiple epidermal growth factor-like domains protein 6	ECM	-
Q96KG7	*MEGF10*	Multiple epidermal growth factor-like domains protein 10	CM	Congenital myopathy 10A, severe variant (CMYP10A) [161]; Congenital myopathy 10B, mild variant (CMYP10B) [162]
A6BM72	*MEGF11*	Multiple epidermal growth factor-like domains protein 11	CM	-
**Q13201**	***MMRN1***	**Multimerin-1** [45,163]	ECM	Factor V Quebec [164]
**Q9H8L6**	***MMRN2***	**Multimerin-2** [45]	ECM	-
Q5VY43	*PEAR1*	Platelet endothelial aggregation receptor 1	CM	Cardiovascular disease [184]
Q15063	*POSTN*	Periostin	ECM	-
A2VEC9	*SSPOP*	SCO-spondin	ECM	-
Q15582	*TGFBI*	Transforming growth factor-beta-induced protein ig-h3	ECM	Corneal dystrophy, epithelial basement membrane (EBMD) [262]; Corneal dystrophy, Groenouw type 1 (CDGG1) [263]; Corneal dystrophy, lattice type 1 (CDL1) [264]; Corneal dystrophy, Thiel-Behnke type (CDTB); Corneal dystrophy, Reis-Bucklers type (CDRB) [265]; Corneal dystrophy, lattice type 3A (CDL3A) [266]; Corneal dystrophy, Avellino type (CDA)
Q5DID0	*UMODL1*	Uromodulin-like 1	CM, CYT	-

Proteins with an EMI domain (annotated in UniProt) are listed. Proteins with experimentally confirmed *O*-fucosylation are highlighted in bold, with references provided. Any known human pathologies associated with listed proteins (reported in UniProt) are indicated with corresponding references. Major subcellular locations for listed proteins (reported in UniProt) are indicated. ECM, extracellular matrix; CM, cell membrane; CYT, cytoplasm.

## Data Availability

Not applicable.

## Supplementary Materials

The following supporting information can be downloaded at: https://www.mdpi.com/article/10.3390/molecules30071470/s1, Table S1: Multiple sequence alignments of FUTs.
